# Investigating the pathways between swift trust and team creativity among nursing student teams in Taiwan: A moderated mediation model

**DOI:** 10.1186/s12912-022-01118-3

**Published:** 2022-12-07

**Authors:** Hsing-Yuan Liu

**Affiliations:** 1 Department of Nursing, Chang Gung University of Science and Technology, Gueishan Township, No. 261, Wunhua 1St Rd, Taoyuan, Taiwan; 2 Department of Nursing, Linkous Chang Gung Memorial Hospital, Taoyuan, Taiwan; 3 Department of Nursing, Chang Gung University, Taoyuan, Taiwan

**Keywords:** Communication, Helping behavior, Trust, Education, Nursing, Taiwan

## Abstract

**Background:**

Considerable theoretical and empirical work indicates that a multitude of factors are associated with team creativity in an organizational context. The complex relationships between the contributors, however, are not well understood in nursing education. This study was to take a process view investigating the pathways from swift trust to creativity via collaborative interactions and to explore whether task conflict would further change the strength of the indirect effect.

**Methods:**

This study utilized a cross-sectional, quantitative, descriptive design. Taiwanese nursing students (final *n* = 629), who enrolled in capstone courses of small interdisciplinary groups collaborating with industrial design students on designing healthcare products, participated in the study. Data were collected from students during 2018 and 2020. Questionnaires assessed their perceptions about teams' swift trust (including cognition- and affect-based), collaborative interactions (including constructive controversy, helping behavior, and spontaneous communication), task conflict, and creativity. SPSS PROCESS macro was used to test the proposed moderated mediation model.

**Results:**

Bivariate correlation analysis showed that greater team creativity was associated with increased cognition-based team swift trust and collaborative interactions. Results revealed that collaborative interactions serving as the underlying mechanisms mediating the effect of cognition- and affect-based swift trust on team creativity. Moreover, the indirect effect of collaborative interactions, specifically, spontaneous communication, on linking swift trust to team creativity varied as a function of task conflict. As task conflict decreased, the effect became stronger.

**Conclusion:**

Findings suggest that nursing student teams’ spontaneous communication serves as the underlying mechanism in linking the relationship between swift trust and team creativity and that lower task conflict plays a crucial role in enhancing the indirect effect. The proposed pathway could provide guidance for nursing educators to promote creativity outcomes by promoting swift trust and collaborative interactions as well as preventing task conflict for interdisciplinary nursing student teams.

## Introduction

Nurses often encounter unexpected situations on a daily basis, such as caring for patients with different health conditions. When nursing routines cannot meet the demands, motivated by the need to improve healthcare outcomes, nurses engage in creative thinking and innovative activities. In pursuing creativity and innovation, teamwork is fundamental to successful nursing care outcomes. Recent studies have shown that innovations are crucial in nursing care and that interdisciplinary collaboration is essential for healthcare teams to generate creative ideas and new problem solutions [1].

Initiating collaboration through the creation of interdisciplinary student teams is a pivotal element of Inter-professional education. When nursing students learn to work collaboratively with other members of a healthcare team they become more competent in delivering comprehensive care for patients [2]. All international healthcare environments can benefit from improved team collaboration. Quantitative assessments of team-level factors associated with successful interdisciplinary collaboration are necessary for determining whether interdisciplinary education can help students work collaboratively [1]. Several factors such as swift trust, teamwork competency, and team creativity have been found to improve the success of interdisciplinary collaboration in business and corporate models designed to enhance swift trust and team creativity. However, these contributors have not been evaluated in nursing education [1].

Interdisciplinary education involves students from two or more disciplines collaboratively learn from each other to facilitate effective cooperation and advance health outcomes [3], which prepares nursing students to working on professionally interdisciplinary healthcare teams [4]. Since 2016, nursing schools in Taiwan have incorporated interdisciplinary capstone courses into their curricula that require teams of students from different departments to generate innovative products that can solve real-world healthcare problems [4].

Team creativity may be fostered by a number of factors and complex relationships exits between the contributing variables. Although considerable attention has been paid to the direct connections between creativity and the related contributors within organizational teams [5], research on the pathways through which teammates build swift trust, engage in collaborative interactions, and develop creativity has been limited. To the best of our knowledge, none of the extant studies has examined pathways where swift trust contributes to collaborative interaction behaviors, which, in turn, exert an effect on team creativity [1]. To fill this knowledge gap, this study took a process view to investigate whether the indirect effect of swift trust on team creativity is mediated through collaborative interaction behaviors and whether this indirect effect is further contingent on different levels of task conflict as perceived by team members.

## Background

### Swift trust as an antecedent to team creativity

Swift trust is the foundation of teamwork. According to Kanawattanachai and Yoo [6], trust takes time to develop because its foundation is based on lengthy, well-established collaborations. In a team context, swift trust is a unique form of collective perception and relationships that can manage issues of vulnerability, uncertainty, risk, and expectations [7]. As a presumptive form of trust, it develops rapidly among group members with a common task and typically in the initial stage of team formation.

Swift trust includes two elements: competency that is cognition based and emotion that is affect based [6]. Cognition-based trust involves individual beliefs about peer reliability and dependability, whereas affect-based trust involves interpersonal care and concern [1]. Greater team trust was found to be associated with greater team creativity [8]. However, the two elements of swift trust may be differentially associated with team creativity. For example, one study found that only cognition-based trust was associated with team creativity, but not affect-based trust [9]. Putting together, one would expect that cognition-based, not affect-based, swift trust is likely to be the antecedent to team creativity.

### Collaborative interactions as underlying mechanisms linking swift trust to team creativity

Several factors such as swift trust, teamwork competency, and team creativity have been found to improve the success of interdisciplinary collaboration in the related business and corporate models. However, these variables have not been evaluated in nursing education [1]. Moreover, components of interdisciplinary team collaborations have been found to be positively related to creativity. For example, Derdowski et al. [10] found that constructive controversy can improve group performance and increase team creativity. Moser et al. [11] suggested that helping behaviors are positively correlated with creative innovation for healthcare teams. McAlpine [12] showed that spontaneous communication among team members can enhance creativity by facilitating information exchange and idea generation.

A supportive team environment is critical to the emergence of creative idea within teams [9]. Cooperative collaborations among team members can lower the fear of disagreement or ridicule, thereby facilitate the generation of novel and divergent input into group products [13]. Upon close examination, collaborative interaction behaviors are multifaceted, including constructive arguments, helping behaviors, and spontaneous communication [13]. Constructive argument or controversy occurs when team members have contrasting ideas or opinions, yet together they seek to reach agreement [14]. A team member’s direct, intentional attempts to help another member with a task that is crucial to the team are deemed to be helping behaviors [15]. Spontaneous communication appears as casual and impromptu interactions between team members encouraging information transfer and idea generation [12]. Each of the above described collaborative interaction behaviors is correlated with increased team creativity [1, 12].

Trusting relationships between team members help create an environment that allows them to interact and communicate regularly and effectively. Swift trust is particularly important when establishing temporary teams [1, 16]. Swift trust has been documented to have a positive effect on constructive controversy, helping behaviors, and spontaneous communication among team members [1, 17]. As team trust is associated with greater team creativity [18, 19], one would expect that swift trust engenders the rapid formation of a collaborative team environment that leads to increased team creativity [20]. In other words, a process is expected to unfold over time, in which team swift trust among teammates leads to enhanced collaborative interactions, which, in turn, lead to increased team creativity. Thus, this study specifically tested the mediating role of collaborative interactions among teammates as an underlying mechanism linking swift trust to creativity.

### Indirect effect conditional on task conflict

Team conflict refers to tension among members due to their real or perceived incompatible goals or interests, which may lead to less than desirable team performance [21]. Three distinct types of team conflict have been identified: task conflict, relationship conflict, and process conflict [22]. Task conflict includes incompatible opinions, views, and perspectives about a task. Relationship conflict emerges when perceived interpersonal mismatches manifest as tension, annoyance, and animosity among team members. Process conflict is defined as disagreements about the logistics of task completion, including roles, responsibilities, and work arrangements [21].

As expected, low levels of team conflict can foster creativity [23]. The effect of team conflict on creativity further depends on the type of team conflict examined. For example, whereas one study found that increased task conflict was associated with greater team creativity [5], another study found that creativity was at highest when task conflict was at a moderate level [24]. By contrast, no empirical studies have reported the effect of relationship and process conflict on creativity. Taken together, it appears that task conflict is particularly crucial to team creativity and that it could alter the strength of the association between team collaborative interactions and team creativity.

The above literature review provided a theoretical foundation for the proposed conceptual framework in this study. Two sets of hypotheses were tested.

**Fig. 1:**
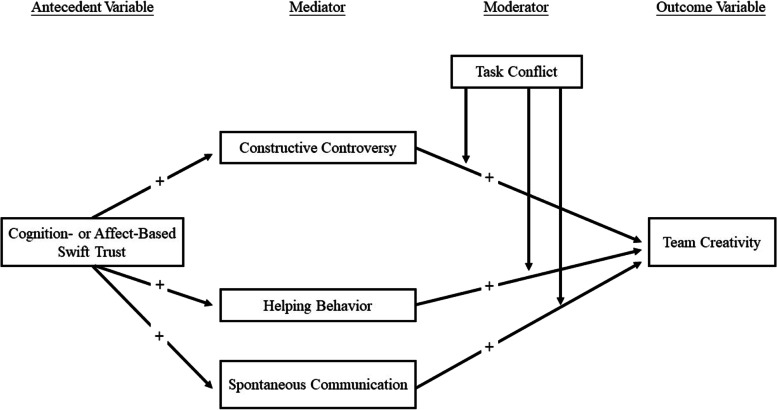
Graphical representation of the m moderated mediation hypothesized pathways

- *Mediation Hypothesis:* A pathway linking swift team trust to team creativity was hypothesized to be fully mediated by collaborative interaction behaviors. In other words, the indirect effect of swift trust on creativity through each dimension of collaborative interactions (i.e., swift trust $\to$ constructive controversy/helping behavior/spontaneous communication $\to$ team creativity) was expected to be without a significant direct effect between swift trust and creativity.
- *Moderated Mediation Hypothesis:* It was hypothesized that the mediating effect of collaborative interactions in the chain-like pathway linking swift trust to team creativity would be contingent on task conflict. Specifically, it was expected that the positive association between collaborative interactions and creativity would be enhanced by lower levels of task conflict (Fig. 1).

## Methods

### Design and Participants

This study employed a cross-sectional, quantitative, descriptive design. A convenient sampling strategy was used for data collection. Taiwanese nursing students (*n* = 650) enrolled in a capstone course of small interdisciplinary groups collaborating with industrial design students on designing healthcare products were recruited to participate in the study. The interdisciplinary capstone course was offered to the nursing students in the last year of their program at a technology and science university in northern Taiwan between January 2018 and January 2020. The major goal of this interdisciplinary capstone course was to help advanced nursing students, who either passed the national nursing licensing exam or completed all-around hospital-based clinical practice, develop the ability to design healthcare products for clinical applications. Offered as a joint course of the nursing program with an industrial design program at another university, a faculty member and several teaching assistants from each of the programs served as the co-instructors. Focusing on the design, making, and testing of a group product, the students from nursing and industrial design programs were randomly assigned into small interdisciplinary teams in the size of 5 to 6 students. In the format of five 4-h in-class workshops, a faculty member presented lectures and teaching assistants supervised small group discussions. Field visits were also scheduled. In addition to the face-to-face interactions during the workshop times, communication and interaction among team members was done in a virtual environment via video conferencing, instant messaging, and phone calls. Students from the industrial design program met in person with the nursing students for three workshops. The first orientation workshop was designed to help students understand the two programs’ specializations and provide instructions on team collaborations and final evaluations on the team projects. The second workshop involved a visit to a design factory and instructions on product prototyping. The final workshop was student presentations on their team products to three experts with varying backgrounds (i.e., clinical nursing, medical engineering, and industrial design) who provided feedback to the students on their prototypes.

Approval was obtained from the Institutional Review Board (IRB) of the hospital ethics committees prior to data collection. Nursing students who attended the last lecture of the interdisciplinary course were provided with a written informed consent form for their voluntary participation in this study. After signing the consent form, they were given a packet identified only by an ID number that contained several questionnaires. A total of 650 packets were distributed and returned at the end of the course. Of the returned packets, 21 were excluded because of incompletion (one or more questions unanswered). The final sample included a total of 629 packets, which were used in this study.

### Instruments

The *Team Swift Trust Scale* was used to assess students’ perceptions of swift trust, a form of collective confidence for a team to start working together. The original scale was developed for multinational teams of students’ in Masters of Business Administration by Kanawattanachai and Yoo [6]. In this study, a Taiwanese version of the scale designed for Chinese populations [17] was used, which included two subscales: cognition-based and affect-based swift trust. Each subscale contains five items. All the statements of items were rated on a 5-point Likert-scale, ranging from 1 for “strongly disagree” to 5 for “strongly agree.” The mean score of items in each subscale was computed separately as the summary index. A higher index score indicated a greater level of swift trust. The original questionnaire’s Cronbach's alphas for cognition- and affect-based components were 0.88 and 0.91, respectively [6]. In this study, the Taiwanese version’s Cronbach's alphas for cognition-based and affect-based swift trust subscales were 0.72 and 0.73 respectively, indicating satisfactory reliability. A factor analysis was performed, which yielded communalities ranging from 0.41 to 0.84 explaining 73.83% of the total variance, suggesting acceptable validity.

The *Team Interaction Behaviors Scale* (TIBS) was modified from an instrument developed by Yang et al. [17] for the Chinese population [25]. The TIBS is a 24-item questionnaire rated on a 5-point Likert-scale, ranging from 1 for “strongly disagree” to 5 for “strongly agree.” This questionnaire assessed three dimensions of collaborative interaction behaviors, including: constructive controversy (4 items), helping behaviors (10 items), and spontaneous communication (10 items). Three summary index scores were computed separately by averaging the items in each subscale of interaction behaviors. A higher index score suggests a greater degree of perceived collaborative interaction. The original instrument for the three interaction behaviors has Cronbach’s alpha coefficients ranging from 0.75 to 0.95 [17]. Cronbach’s alphas of the modified questionnaire used in this study ranged from 0.86 to 0.88, indicating good reliability. The validity of this scale has been demonstrated in a psychometric testing study [25], in which a confirmatory factor analysis yielded communalities ranging from 0.64 to 0.91 explaining 49.27% of the total variance, indicating acceptable validity.

The *Team Conflict Scale* used in this study was adapted from an instrument first developed by Jehn and Mannix [26] and its Chinese version [17] with written permissions. All items were rated on a 5-point Likert-scale, ranging from 1 for “strongly disagree” to 5 for “strongly agree.” In this study only the subscale assessing task conflict was used. A higher score of task conflict suggested a greater degree of heterogeneity in teammates’ views about a task. Cronbach’s alpha for the task conflict subscale was 0.94 in the original instruments and 0.91 in the current study, suggesting good reliability. A factor analysis was conducted, which produced communalities ranging from 0.48 to 0.91 explaining 79.09% of the total variance, demonstrating good validity.

The *Team Creativity Scale* was first developed by Farh et al. [24]. Its Chinese version [17] was used in this and other studies [27]. This 10-item questionnaire employed a 5-point Likert-scale, ranging from 1 for “strongly disagree” to 5 for “strongly agree.” Cronbach’s alpha coefficients reported in previous studies ranging from 0.86 to 0.95 [10]. In this study, the alpha was 0.95, indicating good reliability. The validity of this scale has been demonstrated in a psychometric testing study [25], in which a confirmatory factor analysis produced communalities ranging from 0.77 to 0.89 explaining 49.27% of the total variance, suggesting acceptable validity.

### Data analysis

Data were analyzed using IBM SPSS Statistics version 20.0 (IBM Inc., Armonk, NY, USA). Descriptive statistics were used to evaluate participants’ demographic characteristics and perceptions of collaborative interaction behaviors, team swift trust, team conflict, and team creativity. Pearson’s correlation coefficients were calculated for team-based study variables. The SPSS PROCESS macro [28] based on least-square regression was employed to test the hypothesized pathways. Specifically, a parallel multiple mediator model (Model 4) was applied to test the mediation hypothesis and Model 14 was applied to test the moderated mediation hypothesis. Two sets of analysis were run separately for each hypothesis. Whereas cognition-based swift trust served as the antecedent variable in one model, affect-based swift trust served as the antecedent variable in the other. In all analysis, team creativity served as the outcome variable, the three dimensions of collaborative interactions as the mediators, and task conflict as the moderator. Inference about the indirect effect of antecedent variable on outcome variable through the mediator was assessed by a bootstrapping strategy. A bootstrap confidence interval for the inference about an indirect effect was estimated based on 5000 times resampling. Evidence of moderation of an indirect effect is indicated by a confidence interval for the index of moderated mediation that does not include zero. With evidence of moderated mediation, one can conclude that the chain-like pathway (e.g., cognition-based swift trust → spontaneous communication → team creativity) functions differently according to different levels of task conflict.

## Results

### Descriptive statistics

The majority of the research participants were females (> 80%) and most of the students were in their early twenties (Table 1). Over 90% of the participants expressed satisfaction with the course. Table 1 shows that students rated cognition-based team swift trust higher than affect-based team swift trust. Mean scores for the three dimensions of collaborative interactions from the highest to lowest were: helping behaviors, spontaneous communication, and constructive controversy, in that order. On average, the students’ ratings on the presence of team task conflict was relatively low, whereas their perceived team swift trust and team creativity were moderately high.

**Table 1:** Descriptive statistics and mean scale scores of participants (*n* = 629)

Variables	*n* (%)	Mean (SD)	Range
Gender			
Male	104 (16.5)		
Female	525 (83.5)		
Age		21.3 (0.89)	19–27
Team swift trust		3.45 (0.51)	2.10–5
Cognition-based		3.81 (0.70)	1.80–5
Affect-based		3.08 (0.61)	1.60–5
Collaborative interactions		4.04 (0.62)	1.56–5
Constructive controversy		3.94 (0.61)	2–5
Helping behaviors		4.12 (0.70)	1.10–5
Spontaneous communication		4.02 (0.68)	1.20–5
Task conflict		2.92 (0.90)	1.00–5
Team creativity		4.09 (0.68)	1.00–5

### Correlations between team-based study variables

Pearson’s correlation analysis revealed that students’ ratings on team-based variables were all significantly correlated, with the only exception that team creativity was not significantly correlated with affect-based swift trust (Table 2). First, team creativity was highly and positively correlated with all three dimensions of collaborative interactions, suggesting that greater team creativity was associated with higher constructive controversy, helping behavior, and spontaneous communication. Next, higher levels of team creativity were positively correlated with higher levels of cognition-based swift trust, but not affect-based swift trust. Additionally, team creativity was positively correlated with task conflict, indicating that higher team creativity was related to higher task conflict. Finally, the moderate correlations of both cognition- and affect-based swift trust with task conflict were negative, indicating that greater levels of swift trust in both cognition- and affect-based domains were related to lower levels of task conflict.

**Table 2:** Pearson's correlations for capstone team scores: Team swift trust subscales, collaborative interaction subscales, task conflict, and team creativity (*n* = 629)

Variable	1	2	3	4	5	6	7
Team swift trust							
1. Cognition-based swift trust	-						
2. Affect-based swift trust	0.221^**^	-					
Collaboration interactions	-	-					
3. Constructive controversy	0.667^**^-	0.179^**^	-				
4. Helping behaviors	0.589^**^	0.059	0.721^**^	-			
5. Spontaneous communication	0.502^**^	0.002	0.658^**^	0.856^**^	-		
6. Task conflict	-0.279^**^	-0.250^**^	-0.099^**^	0.440^*^	0.158^**^	-	
7. Team creativity	0.485^**^	0.012	0.677^**^	0.840^**^	0.883^**^	0.196^**^	-

^*^ *p* < .05^**^ *p* < .01

### Analysis results for the mediation and moderation hypotheses

#### Mediation analysis

**Fig. 2:**
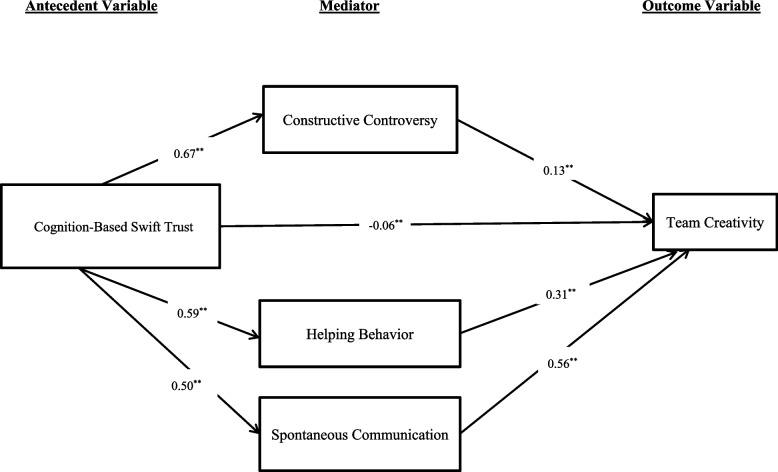
A graphical representation of the mediation analysis results: cognition-based swift trust as the antecedent variable

Two mediation models were tested. In the first model where cognition-based swift trust served as the antecedent variable, analysis results revealed that the indirect effect of swift trust on team creativity mediated through the three dimensions of collaborative interactions, namely, constructive controversy, (Effect = 0.08; Bootstrap 95% Confidence Interval = 0.04—0.12), helping behaviors (Effect = 0.18; Bootstrap 95% Confidence Interval = 0.14—0.22), and spontaneous communication (Effect = 0.28; Bootstrap 95% Confidence Interval = 0.21—0.34) were all significant (Fig. 2). Moreover, the direct effect of cognition-based swift trust on creativity was significant (Effect = -0.06; Bootstrap 95% Confidence Interval = -0.11—-0.02). The total effect was 0.53 (Bootstrap 95% Confidence Interval = 0.46—0.60). Finally, pairwise comparisons were conducted to test the equality of the strength of two specific indirect effects, which showed significant differences between all three pairs of comparisons. Specifically, spontaneous communication demonstrated the largest indirect effect in mediating cognition-based swift trust to creativity, followed by helping behavior and constructive controversy, in that order (Constructive Controversy vs. Helping Behavior: Indirect Effect = -0.104, Bootstrap *SE* = 0.029; Bootstrap 95% Confidence Interval = -0.045 to -0.161; Constructive Controversy vs. Spontaneous Communication: Indirect Effect = -0.199; Bootstrap *SE* = 0.041; Bootstrap 95% Confidence Interval = -0.122 to -0.282; Helping Behavior vs. Spontaneous Communication: Indirect Effect = -0.095; Bootstrap *SE* = 0.045; Bootstrap 95% Confidence Interval = -0.011 to -0.186).

A path diagram in Fig. 2 represents the results of mediation analysis, in which the link of cognition-based swift trust to team creativity is mediated by constructive controversy, a dimension of collaborative interactions. The numerical values are standardized regression coefficients, ^**^ denotes the significance level at 0.01, solid lines (——) represent significant coefficients, and dotted lines (-----) represent non-significant coefficients.

**Fig. 3:**
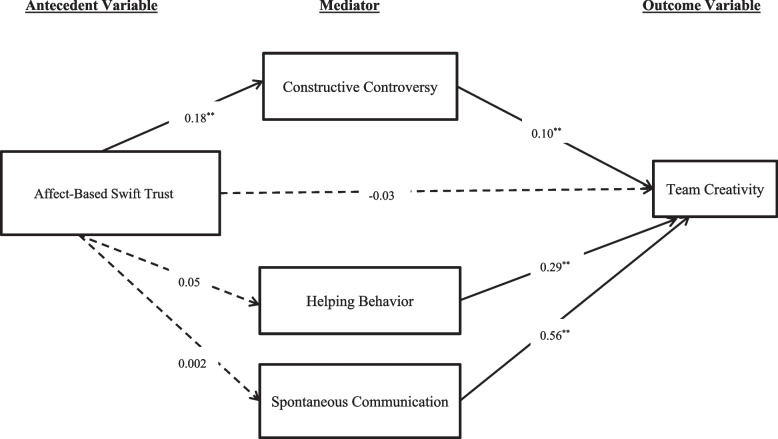
A graphical representation of the mediation analysis results: affect-based swift trust as the antecedent variable

In the second model where affect-based swift trust served as the antecedent variable, analysis results revealed that the indirect effect of swift trust on team creativity through collaborative interactions was only significant for constructive controversy (Effect = 0.02; Bootstrap 95% Confidence Interval = 0.01—0.03), but not helping behaviors (Effect = 0.02; Bootstrap 95% Confidence Interval =  − 0.00—0.04) or spontaneous communication (Effect = 0.00; Bootstrap 95% Confidence Interval =  − 0.05—0.05) (Fig. 3). Overall, the total effect was not significant (Effect = 0.04; Bootstrapping 95% Confidence Interval =  − 0.04—0.11). Finally, pairwise comparisons were conducted to test the equality of the strength of two specific indirect effects, which showed no significant differences between any of the three pairs of comparisons.

A path diagram in Fig. 3 represents the results of mediation analysis, in which the link of affect-based swift trust to team creativity is mediated by constructive controversy, a dimension of collaborative interactions. The numerical values are standardized regression coefficients, ^**^ denotes the significance level at 0.01solid lines (——) represent significant coefficients, dotted lines ( –-) represent non-significant coefficients.

Putting together, the results summarized above provided a partial support for the mediation hypothesis.

#### Moderated mediation analysis

**Fig. 4:**
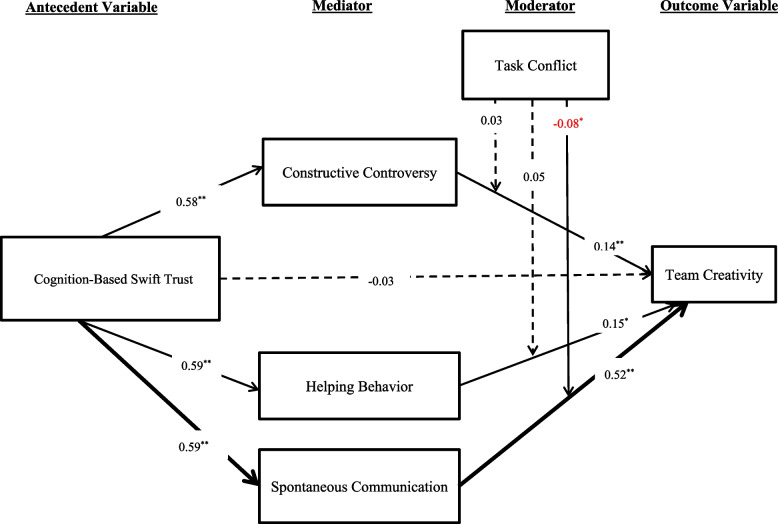
A graphical representation of the moderated mediation analysis results

Two moderated mediation models were tested. In the first model where cognition-based swift trust served as the antecedent variable, analysis results revealed that the indirect effect of swift trust on team creativity through collaborative interactions as a function of different levels of task conflict was only significant for the dimension of spontaneous communication, but not constructive controversy or helping behaviors (Fig. 4). Specifically, as the level of task conflict decreased, the strength of the indirect effect became stronger (Tables 3 & 4).

**Table 3:** Moderated mediation analysis: Cognition-based swift trust as the antecedent variable (*n* = 629)

Model variables	*β*	*SE*	95% CI	*R*^2^
Mediator variable model				
Constant	-2.22^**^	0.10	[-2.423, -2.026]	0.445^**^
Cognition-based swift trust → Constructive controversy (CC)	0.58^**^	0.03	[0.532, 0.634]	
Constant	-2.25^**^	0.03	[-2.491, -2.000]	0.347^**^
Cognition-based swift trust → Helping behavior (HB)	0.59^**^	0.03	[0.525, 0.652]	
Constant	-1.87^**^	0.13	[-2.131, -1.616]	0.252^**^
Cognition-based swift trust → Spontaneous communication (SC)	0.59^**^	0.03	[0.525, 0.652]	
Dependent variable model				
Constant	4.22^**^	0.09	[4.033, 4.401]	0.826^**^
Cognition-based swift trust → Team creativity	-0.03	0.02	[-0.080, 0.015]	
Constructive controversy → Team creativity	0.14^**^	0.03	[0.084, 0.203]	
Helping behavior → Team creativity	0.30^**^	0.04	[0.228, 0.369]	
Spontaneous communication → Team creativity	0.52^**^	0.03	[0.459, 0.590]	
Task conflict (TC) → Team creativity	0.07^**^	0.01	[0.041, 0.098]	
CC x TC → Team creativity	0.03	0.03	[-0.030, 0.087]	
HB x TC → Team creativity	0.05	0.04	[0.003, 0.127]	
SC x TC → Team creativity	-0.08^*^	0.01	[-0.151, -0.014]	

***CI* = **95% confidence interval with the lower and upper limits; *SE* = standard error

^*^p < .05

^**^p < .01

**Table 4:** Moderated mediation analysis: affect-based swift trust as the antecedent variable (*n* = 629)

Model variables	*β*	*SE*	95% CI	*R*^2^
Mediator variable model				
Constant	-0.55^**^	0.12	[-0.794, -0.309]	0.032^**^
Affect -based swift trust → Constructive controversy (CC)	0.18^**^	0.04	[0.102, 0.256]	
Constant	-0.21	0.14	[-0.491, 0.072]	0.004
Affect-based swift trust → Helping behavior (HB)	0.07	0.05	[-0.022, 0.157]	
Constant	0.01	0.14	[-2.282, -2.270]	0.000
Affect -based swift trust → Spontaneous communication (SC)	0.002	0.04	[-0.086, 0.090]	
Dependent variable model				
Constant	4.10	0.06	[-0.042, 0.036]	0.825^**^
Affect -based swift trust → Team creativity	-0.003	0.02	[-0.042, 0.036]	
Constructive controversy → Team creativity	0.13^**^	0.03	[0.073, 0.186]	
Helping behavior → Team creativity	0.29^**^	0.04	[0.219, 0.357]	
Spontaneous communication → Team creativity	0.52^**^	0.03	[0.459, 0.591]	
Task conflict (TC) → Team creativity	0.08^**^	0.01	[0.048, 0.103]	
CC x TC → Team creativity	0.02	0.03	[-0.034, 0.083]	
HB x TC → Team creativity	0.05	0.04	[-0.033, 0.126]	
SC x TC → Team creativity	0.08^*^	0.03	[-0.144, 0.008]	

***CI* = **95% confidence interval with the lower and upper limits; *SE* = standard error

^*^p < .05

^**^p < .01

A path diagram in Fig. 4 represents the results of moderated mediation analysis, in which the link of cognition-based swift trust to team creativity is mediated by the three dimensions of collaborative interactions, namely, constructive controversy, helping behavior, and spontaneous communication. The indirect effect via spontaneous communication is further moderated by task conflict. The numerical values are standardized regression coefficients, ^*^ and ^**^ denote the significance levels at 0.05 and 0.01, respectively, solid lines (——) represent significant coefficients, dotted lines ( –-) represent non-significant coefficients, and thick solid lines (**——**) represent the significant moderated mediation effect.

Taken together, the results summarized above provided a partial support for the moderated mediation hypothesis (Table 5).

**Table 5:** Conditional indirect effects of task conflict (TC): Cognition- or affect-based swift trust as the antecedent variable (*n* = 629)

			Antecedent Variable			
Mediator	Cognition-Based Swift Trust			Affect-Based Swift Trust		
Constructive controversy	Effect	*Boot* 95% CI	*Boot SE*	Effect	*Boot* 95% CI	*Boot SE*
TC low (-1 *SD*)						
TC mean	0.07	[0.011, 0.131]	0.03	0.02	[0.002, 0.042]	0.01
TC high (+ 1 *SD*)	0.08	[0.048, 0.123]	0.02	0.02	[0.010, 0.039]	0.01
Index of moderated mediation	0.10	[0.058, 0.141]	0.02	0.03	[0.013, 0.045]	0.01
Helping behavior	0.02	[-0.022, 0.055]	0.02	0.004	[-0.007, 0.016]	0.01
TC low (-1 *SD*)						
TC mean	0.15	[0.084, 0.209]	0.03	0.02	[-0.003, 0.036]	0.01
TC high (+ 1 *SD*)	0.18	[0.134, 0.217]	0.02	0.02	[-0.004, 0.046]	0.01
Index of moderated mediation	0.20	[0.147, 0.255]	0.03	0.02	[-0.004, 0.048]	0.01
Spontaneous communication	0.03	[-0.014, 0.077]	0.02	0.003	[-0.002, 0.013]	0.004
TC low (-1 *SD*)						
TC mean	0.29	[0.220, 0.379]	0.04	0.001	[-0.052, 0.050]	0.03
TC high (+ 1 *SD*)	0.26	[0.204, 0.317]	0.03	0.001	[-0.045, 0.044]	0.02
Index of moderated mediation	0.22	[0.171, 0.283]	0.03	0.001	[-0.040, 0.038]	0.02
	-0.04	[-0.084, -0.001]	0.02	-0.000	[-0.008, 0.009]	0.004

Boot 95% *CI* Interval = Lower and upper limits of 95% confidence interval of conditional effect as estimated by Bootstrap method

## Discussion

Focusing on the pathways, this study examined the chain-like unfolding process linking swift trust, to collaborative interactions, and then to team creativity, using the data collected from Taiwanese nursing students on interdisciplinary student teams. The antecedent role of two different types of swift trust, including cognition- and affect-based, was investigated, as well as the mediating role of three dimensions of collaborative interactions. First, results from mediation analysis revealed that there was a significant direct effect of cognition-based, but not affect-based, swift trust on team creativity.Conceptualized as a multidimensional construct, the current findings indicated that cognition-based, not affect-based, swift trust exerted a direct influence on team creativity, and, unexpectedly, the direct effect was negative, suggesting higher cognition-based swift trust contributed to lower team creativity. Such finding may explain the inconsistent reports documented in prior research. For example, studies assessed swift trust as a unified variable yielded no significant association with team creativity [29]. When viewed as a multifaceted concept, one study reported that cognition-based, not affect-based, swift trust was positively correlated with team creativity [30], whereas another study reported that neither cognition- nor affect-based swift trust was significantly associated with team creativity [31]. As evidenced by the current findings, the relationship between swift trust and creativity is not a simple and straightforward as previously thought. When investigated through the lens of a nuanced process approach, in addition to the direct effect, an indirect, mediated relationship exists between swift trust and team creativity (see discussion below).

Viewed as a multidimensional construct, collaborative interactions contain constructive controversy, helping behavior, and spontaneous communication. Findings of this study revealed that the indirect effect of cognition-based swift trust on team creativity mediated through the three dimensions of collaborative interaction behaviors were all significant. By contrast, the indirect effect with affect-based swift trust as the antecedent variable was not significant overall. The only significant pathway was mediated through the dimension of constructive controversy. One possible explanation for the differential results could be that Taiwanese nursing students relied more on knowledge sharing (i.e., cognition) than emotional support (i.e., affect) when collaborating on interdisciplinary team projects [1, 32].

Additionally, pairwise comparisons showed that spontaneous communication had the strongest indirect effect, which was followed by helping behavior and constructive controversy, in that order. Students majoring in nursing and industrial design from different schools participated in this study. In addition to the face-to-face interaction time during the course, team members also engage in communication electronically via web conferencing, instant messaging services, and/or phone calls. Perhaps, the combination of the in-class face-to-face interactions and technology-based virtual interactions among teammates was beneficial to spontaneous communication more than helping behavior or constructive [5]. As such the strength of the indirect effect was stronger than helping behavior and constructive communication.

Finally, this study found that task conflict was a significant moderator altering the strength of the indirect effect of cognition-based team swift trust on team creativity through spontaneous communication. Specifically, in the context of lower task conflict, the strength of indirect effect became stronger. Task conflict involves incompatible opinions, views, and perspectives about a task. Spontaneous communication involves individuals communicating unprompted, outside of assigned tasks. Therefore, a higher level of task conflict among team members may create a context where it is prohibitive of the positive effect of spontaneous communication on team creativity [13]. To the best of our knowledge, the current study was the first to investigating the moderating role of task conflict in altering the strength of the indirect effect of swift trust on creativity through collaborative interactions. However, some telltale signs could be found in the research literature. For example, Liang [32] demonstrated that low levels of task conflict enable employees' helping behaviors. Future research needs to further explore other dimensions of team conflict (e.g., process and relationship conflict) and/or characteristics of task (e.g., interdependence) as potential moderators in strengthening or weakening the strength of the indirect effects.

### Implications for interdisciplinary context

The current findings have implications for interdisciplinary teamwork. First, the significant correlations of constructive controversy, helping behaviors, and spontaneous communication with team creativity provide empirical evidence for incorporating interdisciplinary teams in IPE healthcare courses for nursing students. The current findings also provide additional support to the documented reports on the positive relationship between spontaneous communication and team idea generation [12]. Finally, the mediation and moderated mediation models tested in this study could serve as a framework to assist nurse educators when implementing inter-professional education.

### Limitations

In spite of the contributions of findings from this study, it also has some limitations. The first limitation is related to the data collection. Cross-sectional data was collected which could only provide evidence for the correlational, but not causal, relationship between our focal variables. As such the causal pathways revealed in this study cannot be validated. Experimental and longitudinal research designs would be desirable for examining the causality. Second, our research focuses on investigating the effects of swift trust and collaborative interactions on overall team creativity perceived by students, which fails to zoom in on the distinctly different stages of team formation. A contingency model proposed by Farh et al. suggests that moderate levels of task conflict are more likely to be translated into creativity in earlier than later stages of interdisciplinary teamwork [24]. Future research needs to pay more attention on whether the pathways revealed in this study would be applicable to different phases (e.g., early or late) of interdisciplinary team projects. Finally, the study constructs were assessed using only students’ self-reports at one-time point, which might raise the concern about common method bias (CMB). According to Bag [33], the presence of collinearity with variance inflation factors (VIFs) greater than 3.3 indicates a great likelihood of CMB in the model tested. Each model tested in this study was examined for both vertical and lateral collinearities. None of the VIF values for the models exceeded 3.3, providing statistical evidence that the models were free from the CMB problem. To control for CMB, future studies are recommended to employ a cross-time, multi-method design assessing constructs with different research methods (e.g., observing collaborative interactions between teammates) at different time points (e.g., collecting data on swift trust immediately after interdisciplinary teams is formed and assessing team creativity after team projects are completed). Advanced statistical strategies could also be applied to reduce CMB; for example, adding covariates and creating latent variable in model testing.

## Conclusions

To our knowledge, this was the first study using a large sample of nursing students to examine the indirect effect of swift trust on team creativity through collaborative interactions. Our findings suggest that nursing student teams’ collaborative interactions serve as the underlying mechanisms in linking the contribution of swift trust on team creativity and that lower task conflict plays a crucial role in enhancing the indirect effect. The proposed pathways could provide guidance for nursing educators to promote creativity outcomes by promoting swift trust and collaborative interactions as well as preventing task conflict for interdisciplinary nursing student teams. Particularly, relative to constructive controversy and helping behavior, spontaneous communication demonstrated the strongest indirect effect, healthcare educators should encourage a supportive environment in which team members can interact spontaneously and regularly in a face-to-face and virtual environment. Moreover, the findings on task conflict as a significant moderator for the indirect effect also suggest that nursing educators should prevent task conflicts among team members by offering conflict mediation and encourage students building spontaneous communication (e.g., via phone calls, instant messages) into their cooperative structures. Following these steps may maximize the potential for enhancing the creativity of nursing students when formulating interdisciplinary teams.

## Data Availability

The datasets used and/or analyzed during the current study are available from the corresponding author on reasonable request.

## Abbreviations

CMB: Common Method Bias;
TIBS: The Team Interaction Behaviors Scale
